# Hunter–gatherer genetics research: Importance and avenues

**DOI:** 10.1017/ehs.2024.7

**Published:** 2024-02-15

**Authors:** Cecilia Padilla-Iglesias, Inez Derkx

**Affiliations:** Department of Evolutionary Anthropology, University of Zurich, Zurich, Switzerland

**Keywords:** hunter–gatherers, genetics, population genetics, hunter–gatherer ethics, human evolution

## Abstract

Major developments in the field of genetics in the past few decades have revolutionised notions of what it means to be human. Although currently only a few populations around the world practise a hunting and gathering lifestyle, this mode of subsistence has characterised members of our species since its very origins and allowed us to migrate across the planet. Therefore, the geographical distribution of hunter–gatherer populations, dependence on local ecosystems and connections to past populations and neighbouring groups have provided unique insights into our evolutionary origins. However, given the vulnerable status of hunter–gatherers worldwide, the development of the field of anthropological genetics requires that we reevaluate how we conduct research with these communities. Here, we review how the inclusion of hunter–gatherer populations in genetics studies has advanced our understanding of human origins, ancient population migrations and interactions as well as phenotypic adaptations and adaptability to different environments, and the important scientific and medical applications of these advancements. At the same time, we highlight the necessity to address yet unresolved questions and identify areas in which the field may benefit from improvements.

## Graphical abstract

**Figure:**
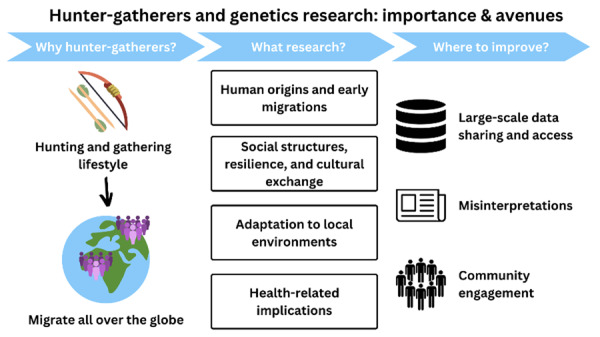

**Social media summary:** Hunter–gatherer genetics research is instrumental for the study of human evolution, but requires reevaluation.

## Introduction

Before the advent of agriculture in the last 10–15 kya (Diamond & Belwood, 2003; Patin et al., 2014), all human populations lived a hunting and gathering lifestyle. It was through this mode of subsistence that members of our species managed to grow in numbers and migrate across the planet. In the present, only relatively few people practise a hunter–gatherer lifestyle, but it is the wide range of geographical and environmental settings they occupy, their reliance on local ecologies and their link to behavioural and subsistence patterns of our ancestors which potentially offer critical insights into the breadth of human behaviour, past and present, and our broader evolutionary origins (Bankoff & Perry, 2016).

The inclusion of contemporary hunter–gatherer communities in genetic studies over the past few decades has contributed significantly to our understanding of the origin of our species, ancient population migrations and interactions, as well as phenotypic adaptations and adaptability to different environments (Fan et al., 2023). Such insights have important implications not only for our understanding of human history, but also of complex phenotypes and diseases, with potential medical applications. While increased genetic representation has yielded important insights and offers important potential benefits, the practice of genetic anthropology is complex and care must be taken in areas such as data sharing and access, and effective and respectful community engagement. Moreover, the isolation of some of these populations and mismatch between their traditional systems of knowledge and Western scientific methods that are often employed by foreign researchers and institutions when performing and interpreting research among hunter–gatherers requires additional ethical considerations. This is particularly the case owing to the vulnerable status of hunter–gatherer societies in an increasingly globalised world, where the integrity of their homelands and social structures is being increasingly compromised in agricultural nation states (Marshall & Marshall, 2007; Marshall et al., 2014). Here we begin with the case for hunter–gatherer genetic research, reviewing how the work has furthered understanding of human evolution, biology, migrations and adaptation, before discussing outstanding questions and identifying areas that may benefit from additional evaluation.

## The importance of hunter–gatherer genetics research

Although hunter–gatherer genetic research has been instrumental in answering several questions regarding the origins and evolution of our species, here we will focus on four particular examples where it has provided unique insights in four domains that are essential for understanding what it means to be human – human origins, social structure, adaptation and health.

### Challenging theories about human origins and early human expansions

A groundbreaking study by Cann et al. in 1987 showed the world how key genetics research among contemporary hunter–gatherers was going to be for understanding the origins of our species, and the processes leading to the genetic diversity observed today. The seminal *Nature* paper revealed that, on average, any two African populations were 50% more distinct from each other in their mtDNA than any two populations from outside of Africa (Cann et al.,^1987^; but see also Johnson et al., 1983 for a similar finding with a larger representative African sample). This suggested a recent migration out of Africa and subsequent expansion, and hence provided grounding for the palaeoanthropological evidence supporting an origin of *Homo sapiens* in Africa (i.e. the ‘Recent Out Of Africa’ hypothesis for the origins of *H. sapiens*) as opposed to a ‘multiregional model’, whereby modern human populations were seen as the result of local evolution in different parts of the world over the past one to two million years (Thorne & Wolpoff, 1981; Stringer & Andrews, 1988; Stringer, 2003; Brauer, 1984; but see Excoffier & Langaney, 1989 and Templeton et al., 1992 for a different interpretation of findings concerning African mtDNA lineages). Nonetheless, when the researchers used the mtDNA sequences to try to reconstruct and date a phylogeny of human populations, they found that the !Kung, a contemporary hunter–gatherer group from Southern Africa, showed consistently the most divergent lineages out of any human population. This also opened up debates about *where* and *when* in Africa our species first arose, as well as about population dynamics and interactions inside the continent throughout our evolutionary history (Meneganzin et al., 2022; see Vicente & Schlebusch, 2022 for a comprehensive review).

Palaeoanthropological and archaeological evidence had traditionally pointed at Eastern Africa as the cradle of humankind because the earliest accepted human skulls had been found in Ethiopia dating more than 200 (233 ± 22) kya (White et al., 2003; McDougall et al., 2005; Vidal et al., 2022). This was around the same age as the estimated 140–290 kya that Cann et al. (1987) had calculated as the date when the most recent matrilineal common ancestor for all modern humans would have lived. It is important to note that the most recent common mtDNA ancestor could post-date or pre-date *H. sapiens* ‘origins’, and depends significantly on effective population size dynamics and the envisaged model of speciation. Nevertheless, similarly aged evidence for the regular use of pigments (276 ± 29 ka to >350 kya; Beaumont and Vogel, 2006), complex symbolic behaviour (~105 kya; Wilkins et al., 2021), marine resources (~164 ± 12 kya; Marean et al. (2007) and other elements considered hallmarks of modern human behaviour had been found too in Southern Africa dating to around the same time (see Meneganzin et al., 2022 for a review, but see also Clark & Brown, 2001; Barham, 2002; Brooks et al., 2018; and Van Peer et al., 2003 for evidence for the collection and regular use of pigments from 320 and 200 ka in central, eastern and northern Africa). Moreover, the appearance of Middle Stone Age technology at Florisbad in South Africa at around 280 kya is of similar age to the cranium found at the same site, which some researchers also interpreted as an early *H. sapiens* (Grün & Beaumont, 2001; Kuman et al., 2020).

However, it was not as simple as switching the location of our species’ origins from Eastern to Southern Africa. Around the same time, hunter–gatherer research from other parts of the continent would prove instrumental for understanding *H. sapiens’* history. For example, in 1969, Cavalli-Sforza and colleagues had studied the role of Central African hunter–gatherers (CAHG) in deep African history (Cavalli-Sforza et al., 1969). Having sampled 175 Aka individuals from the Central African Republic, Cavalli-Sforza and colleagues found that genetic distances based on several genetic markers between African and non-African populations were greater than those between other continents and therefore too suggested an African origin for our species with a split between Africans and Asians around 100,000 years ago (Cavalli-Sforza et al., 1969; Cavalli-Sforza, 1986). They also suggested a common origin for eastern and western CAHG living at opposite ends of the Congo River Basin, almost 3000 km apart (Cavalli-Sforza et al., 1969; Santachiara-Benerecetti et al., 1980). While the exact time depth of the origin of the CAHG lineage and the divergence between eastern and western CAHG would be the subject of subsequent debate for decades (e.g. Bailey & Headland, 1991; Rosas et al., 2022; Hart & Hart, 1986; Bahuchet, 2006, 2012; Lopez et al., 2018; Verdu et al., 2009; Verdu et al., 2013), early mtDNA research also suggested that CAHG could represent a lineage close to the root of the human phylogenetic tree just like the !Kung did (e.g. Cavalli-Sforza, 1986; Chen et al., 2000; Destro-Bisol et al., 2004a, b; Verdu, 2017). Nonetheless, it highlighted the importance of yet another part of Africa in our evolutionary history. In the last four decades, improvements on genetic, bioinformatic and computational resources alongside the sampling of more hunter–gatherer populations from Africa (but also outside of Africa) are helping to fill in the blanks on the processes leading to the emergence of *H. sapiens*.

Whole-genome-sequencing studies including populations representing the three major African hunter–gatherer groups (The Southern African Khoe-San, Eastern African Hunter–Gatherers and CAHG) have inferred deep divergence times between one another, as well as between them and all other humans (Fan et al., 2019; Scheinfeldt et al., 2019; Wang et al., 2020b; Lipson et al., 2022). Based on modelling designed to infer split dates, the lineage leading to the Khoe-San indeed seems basal to all other human lineages with an estimated divergence time of 300–200 kya (Schlebusch et al., 2017; Fan et al., 2019, 2023; Wang et al., 2020a; Bergström et al., 2021; Pfennig et al., 2023). This is followed by the split of CAHG and all other populations (around 200 kya) followed by a split between West and East Africans (around 130 kya) (Schlebusch et al., 2017; Fan et al., 2023). The Hadza and the Sandawe Eastern African Hunter–Gatherers, in turn, would have diverged from non-San/non-CAHG populations between 25 and 60 kya (Fan et al., 2023). Despite these inferred deep divergences, studies have also consistently found evidence for intermittent episodes of gene-flow between all hunter–gatherer lineages (Bergström et al., 2021; Ragsdale et al., 2023). This is important because models are inevitably summaries and approximations of reality and can be thought of as inferring ‘effective’ demographic parameters within their structural constraints, whether of strict population splits or of models with gene flow and admixture (which tend to have higher estimates for initial split dates, e.g. Steinrücken et al., 2019).

Focussing on gene flow, the Baka and Mbuti have been inferred to have split from one another around 50 kya but maintained gene flow during several time windows until the present day (Wang et al., 2020b; Padilla-Iglesias et al., 2022a). The Hadza and the Khoe-San are also inferred to have maintained gene flow until 12 kya, leading to a cline of genetic relatedness between eastern and southern Africa (Fan et al., 2023). Last, private allele sharing between Khoe-San groups and the Mbuti also supports historical gene-flow across south-central Africa (Schlebusch et al., 2020). This is important because even extreme levels of genetic diversity that were previously attributed to archaic admixture may rather (or additionally) be the result of this deep population structure in Africa (Ragsdale et al., 2023; Wang et al., 2020b; see Schlebusch & Jakobsson, 2018; Prendergast et al., 2022; Pfennig et al., 2023; Henn et al., 2018; Hollfelder et al., 2021 for recent comprehensive reviews on genetic insights into human origins in Africa).

The combination of this genetic research among contemporary hunter–gatherers alongside morphological, archaeological and ancient genomic lines of evidence has led to a paradigm shift regarding the origin of our species. Earlier views of a single origin of *H. sapiens* somewhere in east Africa have been replaced with the view that our species originated and diversified within strongly subdivided (i.e. structured) populations, probably living across Africa, that were connected by sporadic gene flow (Stringer, 2016; Scerri et al., 2014, 2018; Ragsdale et al., 2023; Gunz et al., 2009). This concept has been termed the *Structured African Metapopulation model* or *Pan African origin of Homo sapiens*, and explains the observed extreme morphological, cultural and genetic diversity across Africa and over time (Scerri et al., 2019). Moreover, the crucial focus of this model on patterns of interconnectivity and long-range interactions has placed the high rates of mobility and between-group migration that characterise contemporary hunter–gatherer societies at the core of theories of human origins, further highlighting the need to include these populations in human evolutionary research.

In addition to questions regarding the emergence of *H. sapiens*, the routes which early members of our species used to further spread across the globe, which other species we were in contact with, and the number and timing of migrations outside of Africa prompted additional intriguing inquiries (Macaulay et al., 2005). In addition to archaeological evidence (e.g. Lahr & Foley, 1994, 1998; Liu et al., 2015) as well as recent advances in aDNA (e.g. Haber et al., 2016; Kuhlwilm et al., 2016; Reich et al., 2011), genetics research among several extant (semi- or previous) foraging populations has also been vital for our understanding of the patterns of past human migrations and expansions outside of Africa into Eurasia, Oceania and America (Reyes-Centeno, 2016).

Early hypotheses regarding the peopling of Southeast Asia (SEA) relied on phenotypic and ethnographic descriptions of the ethnic groups in the region and had postulated three sequential waves of human migration into Peninsular Malaysia: a first migration by the Semang hunter–gatherers, followed by the Senoi and last the Proto-Malay agriculturalists (Schebesta, 1927). Alternatively, studies based on morphometric and dental analyses of human remains from archaeological sites in mainland SEA had proposed a two-Layer hypothesis (Matsumura et al., 2011; Reich et al., 2011; Jinam et al., 2017). According to this latter hypothesis, mainland SEA would have been first populated by Palaeolithic Hòabìnhian hunter–gatherers followed by Neolithic farmer communities that would have spread from southern China into mainland SEA associated with cereal agriculture (Matsumura et al., 2019).

A study in 2005 compared the mitochondrial DNA (mtDNA) lineages of the Orang Asli, including the Semang who until recently lived as nomadic hunter–gatherers in Malaysia, with a worldwide sample of mtDNA (Macaulay et al., 2005). The finding that these populations carried unique ‘relic’ mtDNA lineages with time depths of up to 63,000 years seemed to confirm archaeological evidence for a continuous occupation of the Malay peninsula by hunter–gatherers from at least 40 kya (and potentially 70 kya; Bulbeck, 2002). Yet the fact that these ‘relic’ lineages seemed to have diverged from the same set of founders as all other non-African populations in the sample led the authors to suggest that all Eurasian groups were the result of a single Out-Of-Africa migration, adding evidence for a single wave of expansion in the peopling of SEA. However, genetic studies from other former hunter–gatherer populations in SEA, such as the Onge and Great Andamanese from the Andaman Islands, had used the distinctness of the ancestry profiles and mtDNA lineages observed in these populations to argue that they would have descended from an earlier Out-Of-Africa dispersal and remained isolated since (Reich et al., 2009; Endicott et al., 2006). Several years later, a genetic study comprising genome-wide single nucleotide polymorphism (SNP) data from 79 Asian populations (but not including the Semang, Onge or Great Andamanese) found support for one major migration out of Africa into SEA, although it could not rule out the possibility of two waves (The HUGO Pan-Asian SNP Consortium et al., 2009).

More recently, and given their distinct genetic profiles, greater efforts to include and integrate more indigenous Malaysian and Thai hunter–gatherers in population genetic analyses have revealed that at least three waves of migration into the Malay peninsula are required in order to explain the observed genetic diversity, with the ancestors of the Semang group of contemporary hunter–gatherers probably representing the first inhabitants of the region (Aghakhanian et al., 2015; Lipson et al., 2018; McColl et al., 2018; Göllner et al., 2022). Likewise, a recent publication affirmed the deep genetic ancestry of several Punan groups living across North Kalimantan, Borneo. It clarified that these groups were not Austronesian derived farmers who underwent a subsistence shift towards foraging, but rather pointed towards a deep pre-Austronesian ancestry and historical isolation on the island (Kusuma et al., 2023). The underrepresentation of indigenous Asian populations and specifically Asian hunter–gatherers in population genetics studies, however, means that the answers to many of the aforementioned questions are still far from conclusive. There is thus a lot more to learn about the migration routes and dispersal times from Africa to Asia and beyond, as well as the connectivity of earlier populations and interactions of modern humans with archaic humans.

### Social structures, resilience and cultural exchange

A key question in archaeology, anthropology and studies of human evolution is whether *observed* cultural changes are the result of the physical migration of people or, alternatively, due to the spread of ideas. In conjunction with ethnographic and ethnohistorical research, genetics studies offer a unique opportunity to address this question. They can also provide insights into why the spread of particular cultural traditions might take one course or another.

For example, the expansion of agropastoralist populations across many parts of the world in the last 5–10 kya has completely changed the demographic and cultural landscapes of many hunter–gatherer communities across several continents (Schlebusch & Jakobsson, 2018; Fortes-Lima et al., 2023; Patin et al., 2017 Wang et al., 2020b; Simões et al., 2023; Scheinfeldt et al., 2019; Lopez et al., 2018; Chakraborty et al., 2020). For example, a whole-genome sequencing study including 34 Asian hunter–gatherer groups showed that 27 out of the 34 groups experienced declines in their effective population size (*Ne*) following the adoption of agriculture in the region (Chakraborty et al., 2020). This was particularly the case for groups inhabiting islands or those who were displaced to isolated forested highlands or plateaus that are not suitable and/or fertile enough for extensive agriculture, such as the Paniyas and Mundas from India or the Senai, Kintak and Kensiu from mainland Malaysia. A similar pattern is found among the Punan groups in Indonesia, who despite showing a signal of deep cultural and genetic ancestry (Lansing et al., 2022; Kusuma et al., 2023) currently harbour unusually long intra-population identity-by-descent segments and runs of homozygosity, indicative of their current geographical isolation.

To look deeper into the consequences of agro-pastoralist expansions on hunter–gatherer populations, Gopalan et al. (2022) combined genetics and archaeology to investigate the demographic consequences of the different responses of hunter–gatherer groups to the expansion of farmers in Eastern Africa. These responses ranged from intermarrying with the incoming group and adopting agropastoralism to adopting agropastoralism but not intermarrying, entering economic-symbolic exchange relationships with the migrant group and moving to ecological regions that were marginal for pastoralism or agriculture. For instance, while groups like the Gumuz who adopted agropastoralism early managed to sustain large effective population sizes, those like the Sandawe or the Majangir, who only adopted agropastoralism in the last 500 years, seemed to have experienced a drastic decline in their *Ne* in the last 60 generations. This was also the case for populations such as the Hadza whose geographical range was restricted to an area unsuitable for cultivation or pastoralism (Blurton-Jones, 2016).

In Central Africa, where the expansion of agriculturalist populations, and in particular of Bantu-speaking peoples, led to the establishment of ‘client–patron’ relationships with local hunter–gatherers (Blench, 1999; Lewis, 2002), the *Ne* values of most of these groups have remained relatively stable despite varying levels of admixture with Bantu farmers (even when calculating *Ne* exclusively with non-admixed genomic segments, as admixture will normally lead to greater genetic diversity and therefore higher Ne estimates; Lopez et al., 2019; Patin et al., 2017; Padilla-Iglesias et al., 2022b). Similarly, while the *linguistic* legacy of CAHG populations has been lost, given that all of them have adopted languages from surrounding farmers (Bahuchet, 2006, 2012), integrating genetic data from these populations with the study of their material culture has revealed that the maintenance of connectivity between them in both ancient and recent times has led to the preservation and exchange of other aspects of culture alongside genes (Fürniss & Bahuchet, 1995; Padilla-Iglesias et al., 2022a, b; Boyette et al., 2022).

Genetics research among hunter–gatherers has also been instrumental in challenging assumptions regarding the lifestyle of these populations, and therefore broadening our understanding of human cultural diversity. For instance, patrilocal marriages, where women move to live with their husband's family, are highly prevalent in most populations worldwide (Seielstad et al. 1998; Destro-Bisol et al., 2004b; Ségurel et al., 2008; Hammer et al., 2001) and had been assumed to be prevalent too among CAHG (see Bahuchet, 1988; Biesbrouck et al., 1999 for a review). However, Verdu et al. (2013) analysed sex-specific patterns of genetic diversity in 23 Central African hunter–gatherer and non-hunter–gatherer populations. Using ABC analyses, they found larger male than female *Ne* in CAHG, while the opposite was true for non-hunter–gatherer groups. This finding is consistent with the fact that although polygynous marriages are prevalent across Africa (and worldwide), they are much rarer among hunter–gatherer societies (Chaudhary et al., 2015; Lewis, 2002; Joiris, 2003). Even more strikingly, sex-specific demographic parameters were consistent with higher female to male migration rates among the non-hunter–gatherers, and the reverse trend for CAHG. Higher male than female migration rates are indicative of patrilocal marriages, yet the genetic findings from CAHG revealed a pattern more closely resembling that of matrilocal populations (Oota et al., 2001; Bolnick et al., 2006; Kumar et al., 2006; Gunnarsdóttir et al., 2011), highlighting the flexibility of hunter–gatherer dispersal patterns (Kramer & Greaves, 2011; Marlowe, 2004; but see Verdu et al., 2013 for alternative interpretations).

While the groups mentioned in the previous section from Africa are all descendants from ancient hunter–gatherers (i.e. are primary hunter–gatherers; Lipson et al., 2022; Bahuchet, 2012), genetic studies have also shed light on how hunting and gathering itself can be a behavioural adaptation to changing socio-economic landscapes. For example, the Ache hunter–gatherers who inhabit the Paraná River plains in Paraguay also speak languages from the Tupi family, that originated as part of a large-scale expansion from Brazil starting around 2 kya and involving populations nowadays practising different types of subsistence activities (Walker et al., 2012; Ferraz Gerardi et al., 2023). Two possibilities have been envisaged regarding their origins. In the first, they would be remnants of the Jê hunter–gatherers who adopted the Guarani language and culture (Walker et al., 2012). In the second, which is also in line with Ache mythology, they would be descendants of a Guarani group who took refuge in the forest and completely lost agricultural skills (Hill & Hurtado, 2017). Although more research is needed, the closer genetic affinity of the Ache with Tupi as opposed to Jê groups has been used to argue for the latter hypothesis (Callegari-Jacques et al., 2008). In Asia, the genetic and linguistic affinities of the Mlabri nomadic hunter–gatherers from Thailand with neighbouring farmer groups suggest that they too most likely have originated from an agriculturalist population (Oota et al., 2005; Stoneking & Delfin, 2010; Xu et al., 2010). More research into the relationship between cultural and genetic evolution among hunter–gatherers is needed to understand the makeup of the incredible population diversity observed around the world throughout our history (Barbieri et al., 2022).

### Adaptation to local environments

Human adaptation studies have predominantly focused on the era following 10,000 years ago, during which most human populations shifted from hunting and gathering to practising agriculture and pastoralism (Fan et al., 2016). This transition led to rapid population growth, increased population densities and a rise in infectious diseases. These relatively recent changes created new selection pressures for adapting to local environments and dietary shifts, resulting in population- or region-specific genetic variations that influence various phenotypes, such as innate immune response, lactose tolerance and fatty acid metabolic efficiency (Evershed et al., 2022; Sabeti et al., 2007; Fumagalli et al., 2015; Fan et al., 2016). However, hunter–gatherers inhabited nearly every terrestrial habitat of the planet thousands of years before the development of agriculture (Kelly, 2013). Hence, hunter–gatherer genomic studies can shed light on important adaptations that might have been key for the dispersal of humans within and outside of Africa into the diverse set of environments they inhabit (see Fan et al., 2016 for an extensive review of local adaptations across human populations).

Africa is an extremely heterogeneous continent, exemplified by its geography and demographic history (Foley, 2018; Scerri et al., 2019). Hence, African hunter–gatherer populations have experienced a heterogeneous mix of selection pressures over the evolutionary history of our species. For instance, the Khomani San have thrived in the Kalahari Desert for thousands of years, even in the face of frequent droughts. They have maintained large effective population sizes, which facilitate adaptive evolution (Sugden et al., 2018; Henn et al., 2011). Researchers have identified distinct variants associated with adiponectin, body mass index and metabolism as potential targets of selection in the San populations (Sugden et al., 2018). The San also exhibit lighter skin compared with other African populations, possibly harbouring unique mutations in genes involved in skin pigmentation and other skin-related traits, including keratin loci (Fan et al., 2023; Martin et al., 2017b). Experimental studies have shown that some of these mutations, such as those within the gene body of *PDPK1* responsible for melanocyte production regulation, lead to reduced skin pigmentation in mice (Scortegagna et al., 2014). Therefore, selection acting on the regulators of this gene seems to have been adaptive in the San population, which lives relatively far away from the equator (within the approximate range of −20 to −30° latitude; Fan et al., 2023).

Another example of how genetics among hunter–gatherers has shed light on the mechanisms and selective pressures driving human phenotypic variation is the short stature that characterises multiple human populations that hunt and gather food in tropical rainforests, including hunter–gatherers in Central Africa (such as the Aka and Mbuti), India (Andaman Islands) and Southeast Asia (such as the Agta, Aeta, and Batak). The convergent origins of this phenotype among hunter–gatherer populations across the world which have independent histories can probably be explained as a response to the similar challenges posed by rainforest or rainforest-edge habitats. This has been confirmed by studies analysing the SNPs potentially responsible for these differences and finding that different genetic architectures are associated with the reduction in body size across hunter–gatherers (Migliano et al., 2013). However, a longstanding debate remains regarding whether short stature represents an adaptive response to food limitation (Shea & Bailey, 1996), high temperature and humidity (Cavalli-Sforza, 1986), structural properties of the forest (e.g. dense vegetation necessitating bent or crouched postures and/or tree climbing for gathering honey; Turnbull, 1986) or instead a consequence of early growth cessation that evolved to facilitate early reproductive onset amid conditions of high adult mortality (Migliano et al., 2007). Recent studies using whole-genome sequencing of multiple CAHG populations have been able to shed light on this debate by providing evidence for strong polygenic selection on height (Lopez et al., 2019; Fan et al., 2023). This suggests that the early reproductive age of Central African rainforest hunter–gatherers may not be the cause of their small body size, but that instead directional selection of height might have resulted in changes in life-history traits because of the pervasive pleiotropy of height-associated genes (Lopez et al., 2019). More research is required to understand the selection pressures behind this phenotype in other parts of the world.

Furthermore, genomic studies offer insights into convergent adaptations resulting from universal pressures associated with the hunting and gathering lifestyle. Selection on genes involved in cardiac function and development has been observed in hunter–gatherer populations in Central Africa, Asia and among the Hadza of Tanzania (Bergey et al., 2018; Fan et al., 2023). Hunter–gatherers dedicate a significant portion of their time to foraging, involving extensive walking in search of resources. Although the distances covered by hunter–gatherers are often not precisely known, studies have indicated that the Mbendjele BaYaka of the Republic of Congo spend 25–50% of daylight hours foraging (Thompson, 2017), and the Hadza of Tanzania average 13 km of daily walking for hunting and gathering activities for men, while women walk 8 km daily for foraging plant foods (Wood et al., 2021). The specific genetic loci associated with cardiac function vary across populations, but the shared aspects of their lifestyles suggest that selection acting on loci involved in heart development could be adaptive in these populations.

Another approach to investigating the adaptive hypotheses underlying genetic variation and the selection pressures arising from the foraging niche involves comparing the genomes of hunter–gatherers who have inhabited rainforest environments for hundreds of thousands of years (e.g. Padilla-Iglesias et al., 2022a) with those of agriculturalist populations who have resided in these environments more recently. For example, Lopez et al. (2019) identified signals of polygenic selection in several Central African hunter–gatherer groups at functions related to the sensing of allergens and microbes and the interaction with viruses, which were absent in neighbouring farmer populations. Highly admixed hunter–gatherer individuals also exhibited an excess of hunter–gatherer ancestry linked to heparin biosynthesis, interleukin production and leukocyte chemotaxis, implying the preferential retention of adaptive hunter–gatherer genetic variations in immune-related functions (Lopez et al., 2019). Similarly, Bergey et al. (2018) found that the cardiac development-related genes under selection among the Ba Twa and Andamanese hunter–gatherers in their study were not under selection among the agriculturalist neighbours of either of these populations.

Together, the results of both studies above point to a long history of adaptation by rainforest hunter–gatherers across the globe to the selection pressures imposed by the high density of pathogens and caloric and nutritional limitations of their environments (Lopez et al., 2019; Bergey et al., 2018). Other hunter–gatherer groups, such as the San hunter–gatherers who live in the Kalahari Desert, where pathogen exposure is more limited, do not show these adaptations, except for those groups that have recently come into contact with other populations. Indeed, a study comparing more isolated San groups with others more in contact with agriculturalists identified several immune genes that may have been targets of strong selection in the latter group, but not in the former. They suggested that contact with other populations may have led to adaptation to the introduced pathogens following increased exposure (Owers et al., 2017). To comprehensively understand the evolutionary drivers behind the varying regions of the genome among populations, further research is necessary, including investigations into the present and past environments inhabited by hunter–gatherers, as well as more extensive sampling within populations and their neighbouring groups (Bergey et al., 2018).

An area of research that has recently received more attention is how hunter–gatherer microbiomes (e.g. the gut and the oral microbiomes) differ those from other populations, in particular populations with whom they share an environment but not a lifestyle (e.g. Dobon et al., 2023; Fragiadakis et al., 2019; Girard et al., 2017; Gomez et al., 2016; Schnorr et al., 2014). Since it is as of yet not quite clear how, in addition to cultural, environmental and geographical factors, host genetics impact the composition and diversity of these various microbiomes and given the focus of this paper on four particular examples, we do not go deeper into this topic (but see Gupta et al., 2017 and Rosas-Plaza et al., 2022 for cross-cultural comparisons of microbiome studies including foraging populations).

### Health-related implications and the genetic architecture of complex traits

Although significant progress has been made in including African hunter–gatherers in various studies, our knowledge regarding genetic variation within and between populations remains relatively limited compared with European populations or indeed other African groups (Popejoy & Fullerton, 2016; Martin et al., 2017a; Sirugo et al., 2019; Fatumo et al., 2022). Several studies have emphasised the need to further pursue these efforts, as understanding the extent of human genetic variation is crucial for reducing current and future health disparities between populations of African and European ancestry but also to improve our understanding of the genetics underlying complex traits (Chaichoompu et al., 2020; Prendergast, 2023).

For example, a recent study that used whole genome sequences of 180 African individuals from 12 different populations discovered over 5 million novel genetic variants, of which 78% were specific to certain populations and many are likely to have functional relevance (Fan et al., 2023). Moreover, from all populations, it was the San and the Central African hunter–gatherers that had the highest number of previously unreported SNPs. Understanding how this population-specific genetic variation influences complex traits not only is important for a better understanding of human biology, but also has implications for health. Technologies relying on identifying risk alleles for predicting the likelihood of several diseases, including cardiovascular diseases, diabetes and cancers, are gaining widespread application in biomedical research and clinical studies (Kullo et al., 2022). One of the most common techniques is the use of polygenic risk scores (PRS), that are calculated by adding risk alleles weighted by effect sizes from genome-wide-association studies. Consequently, for them to offer accurate results, having a representative sample of the overall genetic variation across populations is vital. Indeed, researchers have pointed out the limited applicability of current PRS for individuals of African ancestry given that these are primarily calculated with data from European populations, which harness only a small subset of the genetic diversity observed in Africa (Fatumo & Inouye, 2023; Majara et al., 2023; Privé, 2022).

At the same time, the lower levels of linkage disequilibrium and higher genetic diversity, linked to the large long-term effective population sizes generally found for Africans, observed in African hunter–gatherers, mean that their inclusion in genetics studies also offers significant promise: it provides a larger pool of relevant causal variants and allowing for a more precise identification of these variants (Campbell & Tishkoff, 2010; Jallow et al., 2009; Chaichoompu et al., 2020), which contributes to a deeper comprehension of the genetics underlying complex traits.

This is especially important, since traditional exclusion of African populations, including hunter–gatherers, from genetics research has led to biases in the interpretation of genetics results and of inference based on genetic data (Fatumo & Inouye, 2023; Lachance & Tishkoff, 2013). The lack of representation of the huge genomic diversity present in African populations has led to the inaccuracy of PRS in this continent as well as SNP ascertainment bias as both PRS and SNP chips are calculated and built mostly using individuals of European Ancestry (Fatumo & Inouye, 2023; Lachance & Tishkoff, 2013). For example, when the heterozygosity of variants ascertained in European populations is assessed it can falsely lead to the conclusion that European populations harbour a greater amount of variation than African populations (Eller, 2009) and to misestimates of effective population sizes (Rogers & Jorde, 1996; Lachance & Tishkoff, 2013), providing incorrect reconstructions of the histories of these populations. Using haplotype statistics as opposed to single-locus statistics can reduce the impact of ascertainment bias owing to the higher probability of haplotypes being polymorphic across multiple populations (Conrad et al., 2006; Mamanova et al., 2010; Lohmueller et al., 2009). Other potential remedies have also been proposed (see for instance Lachance & Tishkoff, 2013 or Nielsen & Signorovitch, 2003), but these solutions may not be sufficient for African populations and potentially even less so for the further under-represented hunter–gatherers. Although whole genome sequencing of every individual may not be feasible, if even the newest SNP models prove insufficient (Flegontov et al., 2023), this may be the direction in which future studies must continue to go.

## Discussion

The above clearly demonstrates some of the scientific insights that hunter–gatherer genetic research has offered historically – into the origins of our species and our later demography, the impact of broad social systems on genetic diversity and the consequences of ancient and more recent subsistence transitions, and into aspects of human biological diversity and the evolutionary adaptations underlying that variation, as well as the important potential for more health-related research. However, to answer the vast array of outstanding questions regarding these and other topics, we must address how anthropological genetics involving hunter–gatherer communities is conducted. We summarise some of these issues, including suggestions of how to move forwards.

### Large-scale data sharing and access

The advancement of population genetics has in part been driven by the increasing amount of public data available to researchers. These include the creation of public datasets including hundreds of thousands of individuals from diverse populations (including hunter–gatherers) such as the Simons Genome Diversity Project (Mallick et al., 2016), the Human Genome Diversity Project (Bergström et al., 2020), the Human Origins Affymetrix array (Lazaridis et al., 2016) and the Allen Ancient DNA Resource (Mallick et al., 2023). In addition, the large-scale sharing of data from individual projects through centralised databases such as the European Genome-Phenome Archive has had positive effects. For instance, the democratisation of genetics research through the public accessibility of these public datasets has allowed researchers from places that would normally not have the resources to collect, process and analyse genetic datasets to be able to use genetic data for their work. Although personal information is generally not accessible in these databases and all data are anonymized, small-scale societies such as most hunter–gatherer populations are more vulnerable to the widespread use of these data for myriad research purposes as full anonymization is not as easily achieved (such as would be the case with a sample of 30,000 from the United States).

Supervised access has been proposed as a remedy to mediate this process, ensuring that genetic data cannot be used beyond the scope of intended use as specified by the authors of the original studies. Datasets may also be partly public and partly private, based on the requirements set by the study authors or community (Hudson et al., 2020). As a result, researchers are often asked to submit extensive research proposals involving all collaborators participating in the proposed studies and may even have to submit separate applications for each individual who requires access to the requested dataset. These proposals are reviewed by data access committees that in turn determine whether access to these genomes in question may be granted to the applicants. These committees may comprise various individuals based on what has been agreed upon for a specific project, but in some cases may include members of the participating communities to increase autonomy over populations’ own data. Owing to some of the features of the hunting and gathering lifestyle, including the study community in these committees may hamper the access to datasets. This is because the appointed members of the local communities may be difficult to trace back, resulting in a practical inaccessibility of the data by any research team other than the one who originally collected them. As researchers we should find solutions that allow posterior use of such invaluable (and expensive) genetic data without compromising the security of participants.

### Misinterpretations

Several steps can be taken to avoid the misinterpretations of population genetics findings concerning hunter–gatherers. For example, scientists have recently started calling for a move away from the default clustering of individuals into fixed genetic ancestry categories (that might not reflect the real, continuous structure of genetic variation; Lewis et al., 2022). Grouping individuals into ancestry categories or into groups that are only relevant at certain time points assumes that population labels are homogenous and static through time, which may lead to interpretations that assume that contemporary hunter–gatherers are representatives of palaeolithic individuals (Lewis et al., 2022). Continental ancestry categories as they are used now could be replaced with methods that better capture the continuous nature of genetic variation. Such an approach may entail substituting a single, continental ancestry category with multiple categories based on for instance linguistics, ethnicity and regionality, and ensuring the distinction between environmental and genetic effects when examining variation in disease prevalence.

This could also mitigate some of the potential stigmatisation and discrimination to which even non-participating individuals of study populations are subject to following research publications where group-specific patterns are described (Beskow et al., 2001; Foster et al., 1997; Marshall et al., 2006). This is especially true for small-scale groups and hence many hunter–gatherers owing to existing stereotypes regarding primitivity and socio-economic status (Tsosie et al., 2019). These effects may be further exacerbated by reports from mainstream media, who often sensationalize findings with headlines such as ‘Africa's Pygmies not giving up on their ancient lifestyle’ (Donmez & Bogmis, 2021) and ‘face to face with Stone Age man: the Hadzabe tribe of Tanzania’ (Malone, 2007). Study results may even have an impact on the political position of hunter–gatherers, for instance if they suggest a recent uptake of the hunting and gathering lifestyle or an only recent occupation in the geographical area, which can in turn result in relocation, or forced settlement or otherwise impact land rights (Bankoff & Perry, 2016; Hudson et al., 2020). Contrastingly, research may also help to affirm a population's status as for instance rightful landowners when genetic data can indicate a group's longstanding presence in a certain geographical region, such as may be the case for Kusuma et al. (2023) with the Punan Batu in Indonesia. After their recent recognition as a customary law community by the Bulungan Regency government, research such as that by Kusuma et al. (2023) may aid the community in furthering efforts to protect their homeland, such as obtaining Customary Forest status to stop further mining-caused shrinking of the forests (Arif, 2023; Kusuma, 2023). In general, moving away from ethnic and geographic local ancestral identities may contribute to reducing certain negative outcomes by unlinking research outcomes and ethnicity or group labels, although there are exceptional cases in which populations may indeed benefit from a focus on such identities. The political charge and potential consequences of this research should, however, be deeply considered prior to its onset.

### Community engagement

One way in which we may be able to avoid some of the impact for study communities owing to the misinterpretation of results and the consequences thereof is by including them more closely in the study at different stages of its execution, from its design and testing stages to its evaluation and publication. Such community engagement in a population genetics study might for instance entail that the study population is involved in determining the best way to explain the rationale behind the study. It may also take form of reporting results to the study population prior to publication so that they may have a say in how their results are portrayed towards the outside world (Bankoff & Perry, 2016; Arango-Isaza et al., 2023b). Arango-Isaza et al. (2023a) used this strategy for research with the indigenous Mapuche in Chile and have outlined its value as well as challenges in a recent publication. They detail how to make scientific language more accessible to communities, the importance of local collaborators, amongst other reasons, for cultural translation and as cultural intermediaries, and how to navigate sensitive topics when reporting results to participants, which was done prior to publication. Mangola et al. (2022) highlight the necessity of returning results as well and note that funding agencies such as the National Science Foundation allow researchers to plan for this (see Mangola et al., 2022 for more further details). Marshall and Marshall (2007) propose that active community discussions about the study goals and procedures, for instance with local leaders or through public forums, in turn enhance the understanding of individuals who participate in the studies. With some hunter–gatherer groups, this can be done through the establishment of assemblies where members of the community are encouraged to ask questions and raise concerns regarding the research. This measure has already been enforced in certain cases, such as by the San, who ask for collective permission for study participation to be obtained by the researchers prior to the research (Chennells & Steenkamp, 2018).

Community assemblies can also be useful for establishing points of contact where members of hunter–gatherer populations can return if they have additional questions and for further communication between different parties. Members of mobile hunter–gatherer communities often congregate seasonally in villages or permanent camps, and it is common for a designated chief or responsible person to remain in the permanent settlement all year round as a point of contact between hunter–gatherers living in dispersed camps in the forest (Lewis, 2002; Lieberman et al. 1993; Hill-Tout, 1978; Kelly, 2013). These individuals can in turn more easily access communication points or local collaborators in regular contact with both the research team and the participants. The inclusion of both the study population and local collaborators, and the invaluable contributions of these individuals to our research, should be retributed accurately through co-authorship where relevant but also through further involvement in the research, whereas now they often are not (Haelewaters et al., 2021; Minasny & Fiantis, 2018). This is only one example of benefit-sharing where many more are possible and necessary to build equal relationships, although guidelines and common practices for benefit sharing are still underdeveloped (Mangola et al., 2022). In this manner, assemblies and similar community communication structures can help to build trust and long-term relationships between research communities, collaborators and scientists. Such a foundation is necessary if we want to advance data sharing and improve inclusion and diversity within genomic research, which in turn are pivotal for the advancement of the research itself (Bentley et al., 2017).

### Where to go from here

Despite major advancements in the last 50 or so years, there are still areas of hunter–gatherer genetics which could receive more attention. At this point in time, genome-wide SNP genotypes exist for several hunter gatherer groups and whole genome sequences for a subset of these, mostly from populations in Africa (see Fan et al., 2023 for recent efforts). Increasing genomic coverage for more groups in addition to the sequencing of currently genotyped populations is likely to allow us to answer existing questions regarding the extent of human genetic variation, regarding human origins, geographical dispersals and adaptations to various ecosystems (Ávila-Arcos et al., 2023). Moreover, it will help remove bias from our interpretations by allowing us to build reference panels and estimate genetic parameters (such as recombination or mutation rates) that more accurately reflect human genetic diversity, and that are representative of more populations.

In addition, while population genetics research has traditionally focused on documenting ‘broad strokes’ of continental patterns of migrations and interactions between different populations or large demographic fluctuations, such patterns emerge from the local-scale behaviour of individuals and groups (He et al., 2019; Ávila-Arcos et al., 2023). Consequently, more sampling of individuals within regions and a coupling of genetic and anthropological data are required for understanding the local level processes that give rise to the observed continental level patterns (see Lansing et al., 2022 for examples).

Last, the coupling of data from people who still rely on hunting and gathering as their primary means of subsistence with that of populations that have recently transitioned from a foraging to an agricultural or market-based economy can shed light on finer-scale population history, but also on the consequences of abandoning a hunting and gathering lifestyle on our genomes. That is, it can help us understand present human genetic diversity by shedding light on the selection pressures that human populations worldwide would have experienced following the Neolithic transition (see Owers et al., 2017). These groups include for instance the Chabu of Ethiopia, the Khoekhoe herders of Southern Africa and the Pumé of Venezuela (Gopalan et al., 2022).

## Conclusion

Throughout this paper, we hope to have highlighted the various ways in which the inclusion of extant hunter–gatherers in genetics research has proven vital for our understanding of human evolution, diversity and adaptability to different environments, and moreover, to elucidate the importance of such research for reducing health and other disparities between different populations worldwide. We also hope to have nuanced these ideas by stressing the importance of our continued efforts to improve the way we undertake research as a field and by providing examples of facets in which we believe there is room for growth. Incorporating these propositions in how we perform our studies with hunter–gatherers may very well help us advance the field of anthropological genetics even more than it already has in the last 50 years.

## Data Availability

n/a

## Research transparency and reproducibility

n/a
