# Hunter-gatherer genetics research: Importance and avenues – CORRIGENDUM

**DOI:** 10.1017/ehs.2024.15

**Published:** 2024-04-08

**Authors:** Cecilia Padilla-Iglesias, Inez Derkx

In this Perspective the second corresponding author was missing.

The authors apologise for this error detailed below:

Page 1, Line 5 ‘**Corresponding author.** Cecilia Padilla-Iglesias; Email: cecilia.padillaiglesias@uzh.ch’ should read ‘**Corresponding authors.** Cecilia Padilla-Iglesias; Email: cecilia.padillaiglesias@uzh.ch; Inez Derkx; Email: Inezelisabeth.derkx@uzh.ch’
